# Disseminated Trimethoprim/Sulfamethoxazole–Resistant Nocardiosis in Renal Transplant Recipient, India

**DOI:** 10.3201/eid3211.260963

**Published:** 2026-11

**Authors:** Sandeep Rao Kordcal, Md Tariq Maula, Vivek Malleshappa Suguna, Adil Rashid Khan, Rahul Ranjan, Stanzin Spalkit, Aruna Nambirajan, Gagandeep Singh, Neeraj Nischal, Immaculata Xess, Manish Soneja

**Affiliations:** All India Institute of Medical Sciences, New Delhi, India

**Keywords:** Nocardiosis, *Nocardia*, cotrimoxazole resistance, trimethoprim/sulfamethoxazole, antimicrobial resistance, bacteria, kidney transplantation, opportunistic infections, invasive pulmonary aspergillosis, Linezolid, India

## Abstract

A renal transplant recipient in India receiving trimethoprim/sulfamethoxazole prophylaxis experienced fever, hemoptysis, and neurologic deficits. The diagnosis was pulmonary aspergillosis and disseminated nocardiosis caused by trimethoprim/sulfamethoxazole–resistant *Nocardia*. Antimicrobial drug therapy led to complete clinical and radiological resolution after 6 months. This case highlights a resistant breakthrough *Nocardia* pathogen in a transplant recipient.

Renal transplant recipients on lifelong immunosuppression are at increased risk for opportunistic infections. Nocardiosis affects 0.04%–3.5% of solid-organ transplant recipients; pulmonary cases disseminate in up to 44% of cases, and central nervous system involvement occurs in 20%–44% (*1*,*2*). Concurrent infection with *Nocardia* bacteria and *Aspergillus* fungus accounts for only 5%–7% of posttransplant cases (*2*). Trimethoprim/sulfamethoxazole (cotrimoxazole) has been the mainstay of treatment and prophylaxis, but the rising prevalence of trimethoprim/sulfamethoxazole–resistant *Nocardia* bacteria complicates empirical therapy and underscores the necessity of susceptibility testing for successful outcomes.

Written informed consent for publication of this case report, including clinical details and accompanying radiological and histopathological images, was obtained from the patient; personal identifiers were withheld. Formal ethics committee approval was not required for a single case report according to institutional and journal guidelines.

A 39-year-old woman who was taking tacrolimus, mycophenolate sodium, prednisolone, and trimethoprim/sulfamethoxazole prophylaxis after renal transplant had remained well for 14 months before experiencing fever, hemoptysis, anorexia, and weight loss over 45 days. High-resolution computed tomography of the chest revealed right upper lobe consolidation with pleural effusion (Figure 1, panel A); lung biopsy culture grew *Aspergillus fumigatus*, with a serum galactomannan result of 8.16 optical density index, confirming invasive pulmonary aspergillosis according to 2020 European Organization for Research and Treatment of Cancer and the Mycoses Study Group criteria (*3*). Despite the patient taking voriconazole and liposomal amphotericin B, altered sensorium and bilateral lower-limb weakness developed, leading to a higher center referral.

**Figure 1:**
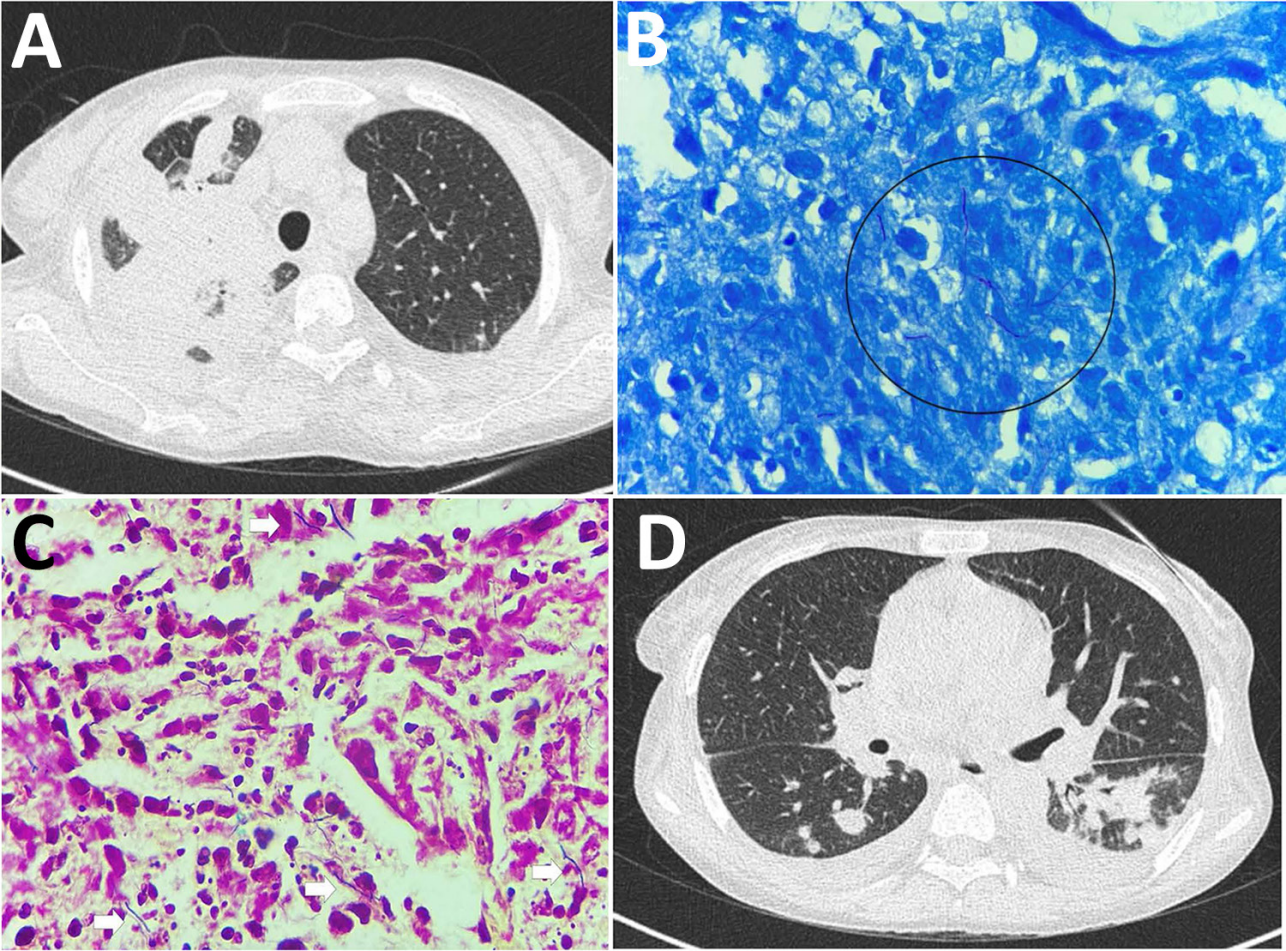
Histopathologic and radiologic findings in renal transplant recipient with disseminated trimethoprim/sulfamethoxazole–resistant nocardiosis, India. A) High-resolution computed tomography of the chest before treatment, showing right-upper-lobe consolidation with adjacent passive atelectasis and a left pleural effusion. B) Lung biopsy, Fite stained, showing weakly acid-fast, branching, beaded filamentous bacteria (circle). Original magnification ×1,000. C) Lung biopsy, hematoxylin and eosin stained, showing suppurative inflammation (arrows). Original magnification ×400. D) High-resolution computed tomography of the chest 6 months after treatment, showing resolution of the consolidation with residual pulmonary fibrosis.

Physical examination revealed asymmetric upper motor neuron weakness in the lower limbs, neck stiffness, and coarse bilateral crackles. Brain imaging revealed multiple ring-enhancing supratentorial lesions with central diffusion restriction and perilesional edema (Figure 2). Repeat transbronchial lung biopsy showed suppurative inflammation with weakly acid-fast, branching, beaded filamentous bacteria when Fite stained and stained with hematoxylin and eosin (Figure 1, panels B and C). Tissue culture on Sabouraud dextrose agar grew dry, chalky colonies suggestive of *Nocardia* spp., although species-level identification could not be confirmed because matrix-assisted laser desorption/ionization time-of-flight mass spectrometry failed twice and 16S rRNA sequencing was unavailable. We interpreted antimicrobial susceptibility by disc diffusion on cation-adjusted Mueller–Hinton agar by using Comité de l’Antibiogramme de la Société Française de Microbiologie thresholds; the organism appeared resistant to cotrimoxazole and ciprofloxacin and susceptible to linezolid, minocycline, imipenem, and amikacin (*4*).

**Figure 2:**
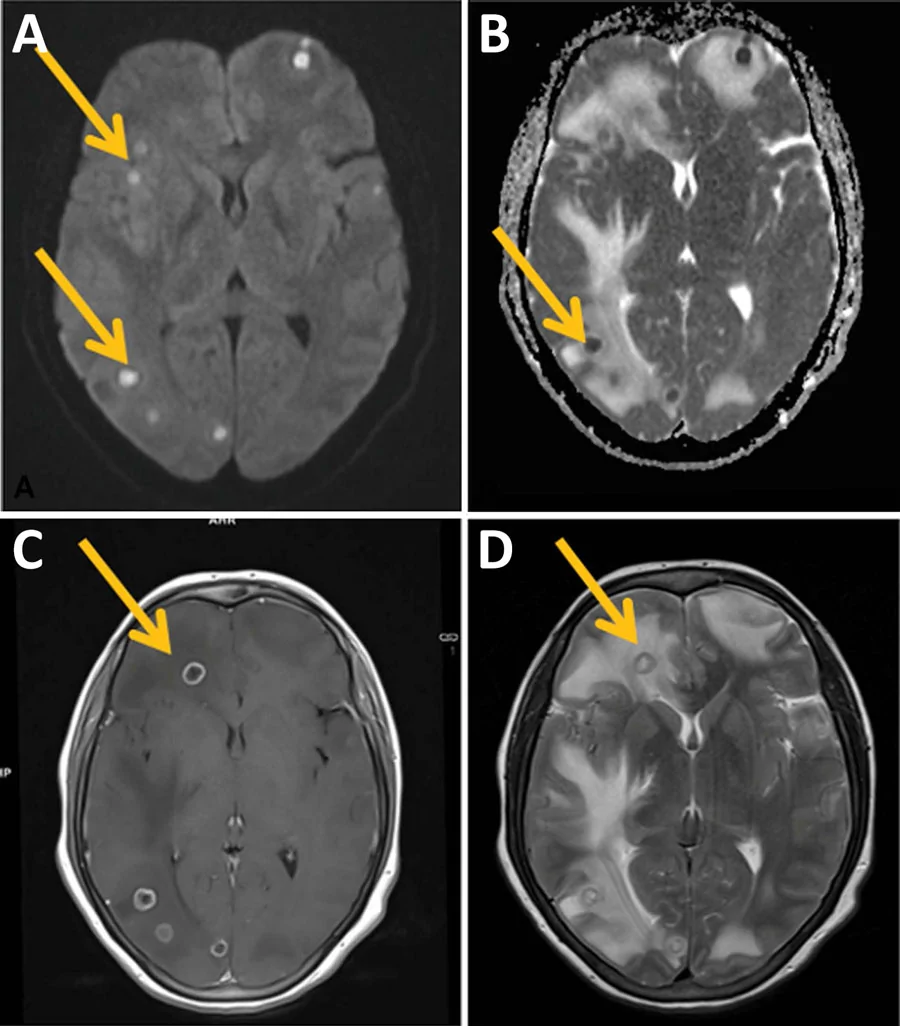
Brain magnetic resonance imaging in renal transplant recipient with disseminated trimethoprim/sulfamethoxazole–resistant nocardiosis, India. A) Diffusion-weighted image showing multiple foci of central diffusion restriction (arrows). B) Apparent diffusion coefficient map showing corresponding signal change (arrow). C) Axial T1-weighted postgadolinium image showing ring-enhancing supratentorial lesions (arrow). D) Axial T2-weighted image showing lesions with central hyperintensity, a hypointense rim, and perilesional edema (arrow).

A diagnosis of disseminated trimethoprim/sulfamethoxazole–resistant nocardiosis with pulmonary and cerebral involvement was made. Tacrolimus and mycophenolate were withheld, low-dose prednisone was continued, and imipenem and linezolid were initiated alongside voriconazole; clinical response was noted within 1 week. After 1 month, cytopenias developed, and linezolid was switched to minocycline. Because of seizure risk, imipenem was changed to meropenem, and the patient was continued on meropenem for 3 months with minocycline. Voriconazole was stopped after 6 months. Imaging at the end of 6 months confirmed radiological resolution in both intracranial and pulmonary lesions, with preserved graft function (Figure 1, panel D). The patient continued to do well at 1-year follow-up and remains on secondary prophylaxis with minocycline.

Trimethoprim/sulfamethoxazole resistance in *Nocardia* bacteria is increasingly reported worldwide, although prevalence varies considerably with isolate source, geographic region, and the susceptibility method used. Susceptibility testing is also technically demanding and might be affected by the lack of control strains of *Nocardia* spp. with known susceptibility patterns. One study found that 42% of United States isolates appear resistant to trimethoprim/sulfamethoxazole and 61% to sulfamethoxazole (*5*), whereas a separate study, comparing 552 isolates across 6 reference laboratories, reported only 2% resistance, attributing the difference in rates to inter-laboratory variability and possible misinterpretation of trailing MIC endpoints (*6*).

Pulmonary infections remain a major cause of illness among postrenal transplant recipients, and opportunistic pathogens such as *Nocardia* bacteria frequently coexist with fungal and mycobacterial disease, with overlapping radiological and clinical features that delay diagnosis in tropical countries. Reported resistance rates among cohorts in India have been of note. In a renal-transplant series of 16 nocardiosis cases, 9 patients were receiving trimethoprim/sulfamethoxazole prophylaxis, and 4 *N. farcinica* isolates were resistant (*7*). In addition, a South India 10-year analysis reported 20.8% resistance among 48 cases (*8*), and a North India center reported nocardiosis in 12 (1.1%) renal transplant recipients, of which 60% isolates were trimethoprim/sulfamethoxazole–-resistant, and 7 of 12 patients (58.3%) had breakthrough infection while receiving prophylaxis (*9*).

Brain imaging is indicated in all cases of pulmonary nocardiosis irrespective of neurologic symptom because of the 20%–44% rate of central nervous system involvement (*1*,*2*). Species-level identification remains a diagnostic challenge, because morphologic and biochemic methods are often inconclusive and the use of matrix-assisted laser desorption/ionization time-of-flight mass spectrometry might be limited (*10*). Disseminated nocardiosis carries a higher mortality rate of 64%–100% among transplant recipients (*2*). This case report underscores the potential for breakthrough nocardiosis caused by trimethoprim/sulfamethoxazole–resistant isolates despite prophylaxis and highlights the importance of antimicrobial drug susceptibility testing to guide targeted therapy and optimize clinical outcomes.
